# Supplementary material for: Unveiling the Bioactive Potential of the Invasive Jellyfish Phyllorhiza punctata Through Integrative Transcriptomic and Proteomic Analyses
Source: Biomolecules. 2025 Aug 4;15(8):1121. doi: 10.3390/biom15081121 (PMC12383608; doi:10.3390/biom15081121)
Supplement: Supplementary file 1 [file biomolecules-15-01121-s001.zip › Suplementary Material/FileS3/PEPFOLD4/XP_065063196_8717/clusters.html]

 PEP-FOLD clusters


Download archive of all models  here   

|  |  |  |
| --- | --- | --- |
| # | file | sOPEP |
| # cluster 1 - Size: 37 |  |  |
|  | XP\_065063196\_8717-model1 | -6.76106 |
|  | XP\_065063196\_8717-model1.1 | -6.76106 |
|  | XP\_065063196\_8717-model1.2 | -6.76106 |
|  | XP\_065063196\_8717-model1.3 | -6.76106 |
|  | XP\_065063196\_8717-model1.4 | -6.76106 |
|  | XP\_065063196\_8717-model1.5 | -6.63503 |
|  | XP\_065063196\_8717-model1.6 | -6.63503 |
|  | XP\_065063196\_8717-model1.7 | -6.63503 |
|  | XP\_065063196\_8717-model1.8 | -6.63503 |
|  | XP\_065063196\_8717-model1.9 | -6.63503 |
|  | XP\_065063196\_8717-model1.10 | -6.3303 |
|  | XP\_065063196\_8717-model1.11 | -6.3303 |
|  | XP\_065063196\_8717-model1.12 | -6.3303 |
|  | XP\_065063196\_8717-model1.13 | -6.3303 |
|  | XP\_065063196\_8717-model1.14 | -5.75668 |
|  | XP\_065063196\_8717-model1.15 | -5.75668 |
|  | XP\_065063196\_8717-model1.16 | -5.75668 |
|  | XP\_065063196\_8717-model1.17 | -5.75668 |
|  | XP\_065063196\_8717-model1.18 | -5.75668 |
|  | XP\_065063196\_8717-model1.19 | -5.75668 |
|  | XP\_065063196\_8717-model1.20 | -5.75668 |
|  | XP\_065063196\_8717-model1.21 | -5.73644 |
|  | XP\_065063196\_8717-model1.22 | -5.73644 |
|  | XP\_065063196\_8717-model1.23 | -5.73644 |
|  | XP\_065063196\_8717-model1.24 | -5.73644 |
|  | XP\_065063196\_8717-model1.25 | -5.70886 |
|  | XP\_065063196\_8717-model1.26 | -5.70886 |
|  | XP\_065063196\_8717-model1.27 | -5.69566 |
|  | XP\_065063196\_8717-model1.28 | -5.67186 |
|  | XP\_065063196\_8717-model1.29 | -5.5285 |
|  | XP\_065063196\_8717-model1.30 | -5.5285 |
|  | XP\_065063196\_8717-model1.31 | -5.30557 |
|  | XP\_065063196\_8717-model1.32 | -5.30557 |
|  | XP\_065063196\_8717-model1.33 | -5.28682 |
|  | XP\_065063196\_8717-model1.34 | -5.26375 |
|  | XP\_065063196\_8717-model1.35 | -4.71258 |
|  | XP\_065063196\_8717-model1.36 | -4.67451 |
| # cluster 2 - Size: 32 |  |  |
|  | XP\_065063196\_8717-model2 | -5.91876 |
|  | XP\_065063196\_8717-model2.1 | -5.91876 |
|  | XP\_065063196\_8717-model2.2 | -5.91876 |
|  | XP\_065063196\_8717-model2.3 | -5.91876 |
|  | XP\_065063196\_8717-model2.4 | -5.91876 |
|  | XP\_065063196\_8717-model2.5 | -5.91876 |
|  | XP\_065063196\_8717-model2.6 | -5.91876 |
|  | XP\_065063196\_8717-model2.7 | -5.91876 |
|  | XP\_065063196\_8717-model2.8 | -5.91876 |
|  | XP\_065063196\_8717-model2.9 | -5.91876 |
|  | XP\_065063196\_8717-model2.10 | -5.91876 |
|  | XP\_065063196\_8717-model2.11 | -5.91876 |
|  | XP\_065063196\_8717-model2.12 | -5.91876 |
|  | XP\_065063196\_8717-model2.13 | -5.91876 |
|  | XP\_065063196\_8717-model2.14 | -5.91876 |
|  | XP\_065063196\_8717-model2.15 | -5.91876 |
|  | XP\_065063196\_8717-model2.16 | -5.91876 |
|  | XP\_065063196\_8717-model2.17 | -5.91876 |
|  | XP\_065063196\_8717-model2.18 | -5.91876 |
|  | XP\_065063196\_8717-model2.19 | -5.91876 |
|  | XP\_065063196\_8717-model2.20 | -5.91876 |
|  | XP\_065063196\_8717-model2.21 | -5.91876 |
|  | XP\_065063196\_8717-model2.22 | -5.91876 |
|  | XP\_065063196\_8717-model2.23 | -5.91876 |
|  | XP\_065063196\_8717-model2.24 | -5.91876 |
|  | XP\_065063196\_8717-model2.25 | -5.91876 |
|  | XP\_065063196\_8717-model2.26 | -5.91876 |
|  | XP\_065063196\_8717-model2.27 | -5.91876 |
|  | XP\_065063196\_8717-model2.28 | -5.91876 |
|  | XP\_065063196\_8717-model2.29 | -5.12923 |
|  | XP\_065063196\_8717-model2.30 | -4.98982 |
|  | XP\_065063196\_8717-model2.31 | -4.98982 |
| # cluster 3 - Size: 28 |  |  |
|  | XP\_065063196\_8717-model3 | -5.75609 |
|  | XP\_065063196\_8717-model3.1 | -5.75609 |
|  | XP\_065063196\_8717-model3.2 | -5.7428 |
|  | XP\_065063196\_8717-model3.3 | -5.7428 |
|  | XP\_065063196\_8717-model3.4 | -5.72221 |
|  | XP\_065063196\_8717-model3.5 | -5.72221 |
|  | XP\_065063196\_8717-model3.6 | -5.7155 |
|  | XP\_065063196\_8717-model3.7 | -5.70999 |
|  | XP\_065063196\_8717-model3.8 | -5.70999 |
|  | XP\_065063196\_8717-model3.9 | -5.70954 |
|  | XP\_065063196\_8717-model3.10 | -5.70954 |
|  | XP\_065063196\_8717-model3.11 | -5.70954 |
|  | XP\_065063196\_8717-model3.12 | -5.70954 |
|  | XP\_065063196\_8717-model3.13 | -5.7075 |
|  | XP\_065063196\_8717-model3.14 | -5.7075 |
|  | XP\_065063196\_8717-model3.15 | -5.34004 |
|  | XP\_065063196\_8717-model3.16 | -5.34004 |
|  | XP\_065063196\_8717-model3.17 | -5.34004 |
|  | XP\_065063196\_8717-model3.18 | -5.34004 |
|  | XP\_065063196\_8717-model3.19 | -5.34004 |
|  | XP\_065063196\_8717-model3.20 | -5.34004 |
|  | XP\_065063196\_8717-model3.21 | -5.33427 |
|  | XP\_065063196\_8717-model3.22 | -5.32696 |
|  | XP\_065063196\_8717-model3.23 | -5.2884 |
|  | XP\_065063196\_8717-model3.24 | -5.27794 |
|  | XP\_065063196\_8717-model3.25 | -5.2516 |
|  | XP\_065063196\_8717-model3.26 | -5.24408 |
|  | XP\_065063196\_8717-model3.27 | -5.17916 |
| # cluster 4 - Size: 3 |  |  |
|  | XP\_065063196\_8717-model4 | -5.67552 |
|  | XP\_065063196\_8717-model4.1 | -5.29497 |
|  | XP\_065063196\_8717-model4.2 | -5.15947 |
|  |  |  |
